# The Role of Exosomal Non-Coding RNAs in Colorectal Cancer Drug Resistance

**DOI:** 10.3390/ijms23031473

**Published:** 2022-01-27

**Authors:** Dimitra Ioanna Lampropoulou, Evangelia Pliakou, Gerasimos Aravantinos, Dimitrios Filippou, Maria Gazouli

**Affiliations:** 1 Second Department of Medical Oncology, General Oncology Hospital of Kifissia “Agioi Anargiroi”, 14564 Athens, Greece; d_lambropoulou@yahoo.gr (D.I.L.); evangeliaplk@hotmail.com (E.P.); garavantinos@yahoo.gr (G.A.); 2 Department of Anatomy and Surgical Anatomy, Medical School, National and Kapodistrian University of Athens, 11527 Athens, Greece; d_filippou@hotmail.com; 3 Laboratory of Biology, Medical School, National and Kapodistrian University of Athens, 11527 Athens, Greece

**Keywords:** extracellular vesicles, exosomes, non-coding RNAs, colorectal cancer, chemoresistance, drug resistance

## Abstract

Background: Colorectal cancer (CRC) is one of the most common types of cancer diagnosed worldwide with high morbidity; drug resistance is often responsible for treatment failure in CRC. Non-coding RNAs (ncRNAs) play distinct regulatory roles in tumorigenesis, cancer progression and chemoresistance. Methods: A literature search was conducted in PubMed database in order to sum up and discuss the role of exosomal ncRNAs (ex-ncRNAs) in CRC drug resistance/response and their possible mechanisms. Results: Thirty-six (36) original research articles were identified; these included exosome or extracellular vesicle (EV)-containing microRNAs (miRNAs), long non-coding RNAs (lncRNAs), circular RNAs (circRNAs) and small-interfering (siRNAs). No studies were found for piwi-interacting RNAs. Conclusions: Exosomal transfer of ncRNAs has been documented as a new mechanism of CRC drug resistance. Despite being in its infancy, it has emerged as a promising field for research in order to (i) discover novel biomarkers for therapy monitoring and/or (ii) reverse drug desensitization.

## 1. Introduction

Colorectal cancer (CRC) is one of the most common types of gastrointestinal cancers and the third most common cancer diagnosed worldwide. More than 1.14 million new cases are diagnosed, and 576,850 deaths are attributed to CRC annually [1]. CRC is characterized by high morbidity and mortality. Improved screening rates have resulted in longer survival for early-stage CRC patients. However, the 5-year survival rate for patients with advanced CRC remains low due to its heterogeneity, metastatic potential and/or delay in diagnosis [2]. Therefore, exploring CRC tumorigenesis and identifying novel molecular markers are keystones in screening, diagnosis, and successful treatment of CRC.

The most common and crucial treatment in controlling CRC progression remains chemotherapy. However, therapy resistance has been a major barrier against successful treatment, especially if the disease is diagnosed in advanced stages. Indeed, although stage IV patients initially benefit from fluorouracil-and platinum-based chemotherapy, most of them develop chemotherapy resistance due to intrinsic or acquired mechanisms [3]. Nevertheless, the exact underlying mechanisms that lead to CRC cell proliferation and chemotherapy resistance remain under investigation.

Two types of drug resistance exist: intrinsic and acquired. The intrinsic type is characterized by cancer cells exhibiting preexisting resistance against medications before the initiation of treatment; more specifically, tumor cells alter the transportation of a drug, degrade it, and affect the interaction between the drug and its molecular target. On the other hand, in acquired drug resistance, the population of cancer cells has already received the drug and has become resistant in a later stage, leading to tumor growth and proliferation [4].

Exosomes are a class of small extracellular vesicles (EVs, ~30–150 nm diameters) which are being released via an endocytic pathway in almost all mammalian cell types. In recent years, they have evolved as promising biomarkers and therapeutic targets in plenty of chronic illnesses, including cancer [5]. Exosomes seem to serve as crucial mediators of the tumor microenvironment (TME) heterogeneity and have a significant role in the transportation of essential cargos, including cancer-related signaling molecules. The latest can be transferred to other cells via fusion of the exosomes with the target–cell membrane. Upon internalization, exosomes unload their cargos inside the recipient cells. This content includes metabolites, proteins, lipids, nucleic acids, noncoding RNAs (ncRNAs), DNA, and even mRNAs. Thus, the unique exosomal ncRNA signatures may serve as important biomarkers for the early diagnosis and prognosis of various cancer types [6].

NcRNAs (microRNAs (miRNAs), small interfering RNAs (siRNAs), antisense RNAs (asRNAs), circular RNAs (circRNAs), piwi-interacting RNAs (piRNAs) and long non-coding RNAs (lncRNAs)) represent a class of functional RNA with distinct regulatory effects in tumorigenesis and cancer progression. Over the last decade, the role of ncRNAs has been an area of extensive interest across several diseases. Cancer-oriented studies have investigated their dysregulation in malignant proliferation, metastasis, chemoresistance, and inflammatory response [5,7,8,9,10]. To date, miRNAs and lncRNAs represent the exosomal ncRNA subtypes that have been studied the most.

It has been suggested that exosomes transfer nucleic acids from drug-resistant to drug-sensitive cells, expanding the resistance ability among cancel cells. Currently, an increasing number of studies are investigating the effects of exosomal ncRNAs on CRC drug resistance [11]. On the other hand, exosomes seem to possess many desirable features of an ideal drug delivery vehicle, such as biological barrier permeability, long circulating half-life, biocompatibility, minimal immunogenicity, and toxicity, as well as intrinsic targeting capability [10]. Thus, research has also focused on successfully formulating EVs in order to deliver various therapeutics, such as doxorubicin in lung cancer [12,13], and miRNAs, such as miR-30e in cholangiocarcinoma [14]. Moreover, Ha et al. suggested that the combined delivery of selected miRNAs and chemotherapy drugs may be a promising way to overcome drug resistance in cancer [15].

Despite that there are already few reviews regarding the role of exosomal ncRNAs in CRC, none summarizes the role of all exosomal ncRNA subtypes in CRC drug resistance. Hence, our aim is to sum up and discuss the functions of exosomal ncRNAs in CRC drug resistance and response. For clarity, we have included data that refer to both “exosomes” and “EVs”, since these terms often coincide in existing literature.

## 2. Methods and Results

**Figure 1:**
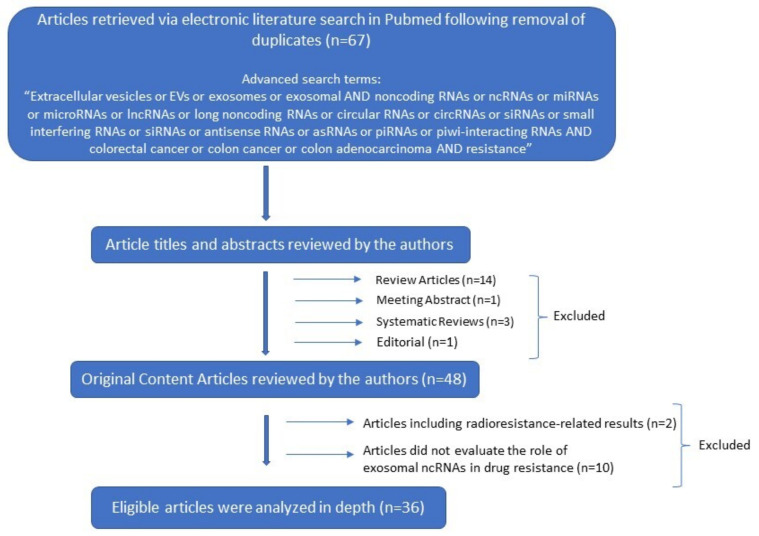
Decision tree for literature research strategy.

A literature search was conducted in PubMed database as of 26 December 2021, using the following terms: “Extracellular vesicles or EVs or exosomes or exosomal AND noncoding RNAs or ncRNAS or miRNAs or microRNAs or lncRNAs or long noncoding RNAs or circular RNAs or circRNAs or siRNAs or small interfering RNAs or antisense RNAs or asRNAs or piRNAs or piwi-interacting RNAs AND colorectal cancer or colon cancer or colon adenocarcinoma AND resistance”. There were no language restrictions, since all articles were in English. Further screening excluded duplicate articles that were found during the above-mentioned advanced search. From the 67 publications, 48 were original content, 14 review, 3 systematic review articles, 1 meeting abstract and 1 Editorial. Only the original research articles were included. Consequently, the titles and abstracts of the original content articles were evaluated for their relevance for the current review, before downloading the full texts. Further screening excluded 2 original articles which announced radioresistance results. We finally identified 36 original research articles that were appropriate for the current work. Two independent investigators (EP and DIL) reviewed and filtered the titles, abstracts, and full texts of the related articles (Figure 1).

A total of 23 out of 36 identified original studies included exosome or EV-containing miRNAs, 6 out of 36 studies were associated with exosome or EV containing lncRNAs whereas 5 and 2 studies included exosomal circRNAs and siRNAs respectively. No studies were found for piwi-interacting RNAs. The majority of them was conducted both in vitro and in vivo and they presented pre-clinical and clinical data. The drugs under investigation were 5-fluorouracil (5-FU, nine studies), oxaliplatin (OX, sixteen studies), their combination (or FOLFOX-like regimen, six studies), doxorubicin (DOX, one study), mitomycin (one study), methotrexate (MTX, one study), irinotecan (IRI, one study), immune checkpoint inhibitors (ICIs, one study) and cetuximab (one study).

## 3. Biological Mechanisms of Exosomal ncRNAs in CRC Drug Resistance

Resistance to anticancer therapies has been a topic of great scientific interest and extensive investigation. Over the years, several chemoresistance mechanisms have been identified including (i) decreased intracellular drug concentrations, via drug efflux transporters, such as P-glycoprotein (P-gp); (ii) DNA damage, cell cycle-mediated and apoptosis-related mechanisms; (iii) altered drug metabolism; (iv) alteration of the drug target; (v) multi drug resistance (MDR); and (vi) epigenetic mechanisms including DNA methylation and histone alterations [16].

**Figure 2:**
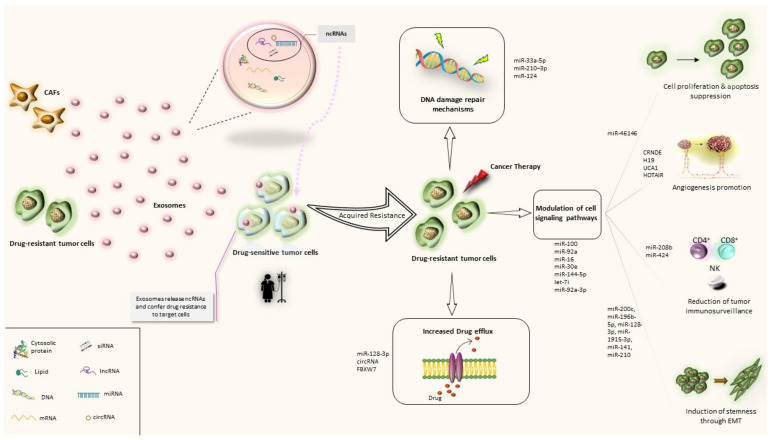
Exosomal ncRNA-related mechanisms implicated in CRC drug resistance. CAFs: Cancer associated macrophags; ncRNAs: noncoding RNAs; miRNA: microRNA; circRNA: circular RNA; lncRNA: long non-coding RNA.

Since TME has been suggested to play a critical role in treatment resistance, exosome-mediated cell communication has been proposed as a novel mechanism of drug resistance. Over the past few decades, several exosome-related mechanisms underlying cancer drug resistance have been described. The first route has been associated with the direct export of the drugs following exosomal incorporation. This theory has been first implied by Shedden et al., when they suggested that doxorubicin could be ejected into the extracellular fluid via vesicle shedding [17]. Moreover, exosomes can regulate drug resistance via horizontal transfer of drug efflux pumps to sensitive cancer cells. Among various drug transporters (such as multidrug-resistant protein-1 (MRP-1), adenosine triphosphate (ATP)-binding cassette transporter 2 (ABCA-2) and adenosine triphosphate (ATP)-binding cassette transporter 3 (ABCG-3)), P-gp (permeability glycoprotein) has been studied the most; exosome-mediated drug efflux pump transfer has been shown to generate the drug-resistant phenotype to drug-sensitive cells, contributing to the development of acquired drug resistance [18,19,20,21,22,23]. Furthermore, cancer stem cells (CSCs) are suggested to be responsible for tumorigenesis and chemoresistance; the WNT signaling pathway has been also linked with the maintenance of stemness in CRC. A number of studies have reported that exosomes derived from fibroblasts and cancer-associated fibroblasts (CAFs) stimulate stemness capacity and contribute to CRC chemoresistance via reprogramming of differentiated cells to CSCs [3,24,25,26]. Finally, the most studied mechanism for exosome-mediated drug resistance refers to the exosomal transfer of bioactive cargo. The latest includes RNA, DNA and proteins and is transported from (a) drug-resistant tumor cells or (b) stromal cells (such as CAFs) to drug sensitive cancer cells. Reportedly, this kind of exosome-mediated interaction plays an important role in conferring drug resistance phenotype to a drug sensitive cancer cell. In the following paragraphs we will discuss the resistance-related mechanisms that are associated with exosomal ncRNAs, focusing on CRC and/or CRC-approved drugs (Figure 2).

Among the diverse exosomal RNA species, miRNAs are the most abundant [27] and therefore more studied. Increasing evidence supports the selective exosomal packaging and transfer of drug-resistance-associated miRNAs [28,29,30]. CAFs are known to promote epithelial-mesenchymal transition (EMT), stemness, invasion, metastasis, and resistance of CRC cells. CAFs function by directly transferring exosomes to CRC cells, leading to a significant release and increase of ncRNA levels. For example, CAF-mediated miR-92a-3p transfer has been implicated with 5-FU/OX resistance in CRC [3]. Exosomal miRNA-induced drug resistance has been also associated with signaling pathways, such as PTEN/protein kinase B (AKT)/phosphoinositide 3-kinases (PI3K). It is well established that loss of PTEN expression, which is a tumor suppressor and negative regulator of PTEN/AKT/PI3K pathway, is involved in CRC carcinogenesis and drug resistance [31]. Indeed, macrophage-derived exosomal miR-223 has been correlated with enhanced drug resistance, via the PTEN/AKT/PI3K pathway, in ovarian cancer [32]. Furthermore, modulation of KRAS signaling pathway has been described as another possible molecular mechanism of drug resistance. Studies including cetuximab have shown that KRAS status is associated with epidermal growth factor receptor (EGFR) pathway, miRNAs synthesis and their sorting into exosomes [33]. Additionally, exosomal miRNAs have been reported to contribute to CRC chemoresistance by regulating EMT and CSCs [34]. EMT and CSC phenotypes favor the EV-producing phenotype of cancer cells and tumor-released EVs promote EMT and stemness in recipient cells [35]. Another exosomal miRNA-related chemoresistance mechanism includes tumor-associated macrophages (TAMs). TAMs are critical components of the TME, and their accumulation has been associated with poor prognosis. In general, TAM inhibition can reverse chemotherapy resistance both in vivo and in vitro [36,37]. Despite that TAMs resemble M2-polarized macrophages, they can exhibit phenotypes anywhere between M1 and M2 polarized states. On this basis, there is evidence that ex-miRNAs can modulate TAM phenotypes and thus reverse drug resistance, i.e., miR-770 which seems to enhance drug sensitivity to doxorubicin in triple-negative breast cancer [38]. Ex-miRNAs can also be secreted by TAMs; Binenbaum et al. reported that miRNA-365 secreted by M2-phenotype TAMs promotes gemcitabine resistance in pancreatic ductal adenocarcinoma [39]. Recently, HER-2 targeted therapy (trastuzumab and pertuzumab) has gained approval for HER-2 positive metastatic colorectal cancer (mCRC) patients. Han et al. found that miR-567-loaded exosomes can increase trastuzumab sensitivity in breast cancer patients, via targeting a carcinogenesis associated protein, autophagy-related protein 5 (ATG5) [40].

While miRNAs exert their function post-transcriptionally by regulating the expression of proteins, via targeting mRNAs, lncRNAs usually act as a competing endogenous RNA (ceRNA) of miRNAs to modulate drug resistance. For instance, exosome-transferred lncRNA H19 has been identified as a ceRNA of miR-141, promoting oxaliplatin resistance in CRC [41]. Ex-lncRNAs have been also reported to promote drug resistance on the translational level, by interacting with RNA-binding proteins. For example, exosomal transfer of the lncRNA CCAL from CAFs to CRC cells results in oxaliplatin resistance, via the post-transcriptional regulation of an RNA-binding protein ((human antigen R (HuR)) [42]. Similarly, exosomal MALAT-1 sponges miR-26a/26b (via the PI3K/Akt/mTOR axis) and regulates several cellular and molecular activities involved in tumor formation, invasion, metastasis and chemoresistance [43]. Along with lncRNAs, circRNAs also represent a reservoir of potential miRNA targets since they can act as miRNA sponges and bind with mature miRNAs. Several studies have reported that circRNAs can promote drug resistance through sponging miRNAs. For example, ex-circNFIX has been found to confer temozolomide resistance to sensitive cells, by sponging miR-132, in glioma [44]. Recently, another ex-circRNA was associated with oxaliplatin resistance in CRC. Interestingly, Wang et al. hypothesized that increased intracellular ATP could result in enhanced capacity of the drug efflux pumps (such as P-gp) to export drugs. Consequently, he found that exosomal hsa_circ_0005963 promoted oxaliplatin resistance by sponging miR-122, thereby promoting glycolysis and PKM2 (M2 isoform of pyruvate kinase) upregulation [45].

Anti-angiogenic agents are widely used as a treatment strategy for mCRC. However, resistance towards anti-angiogenic drugs compromises disease control and often leads to little benefit. Tumor-secreted exosomes have been implicated in cancer angiogenesis by reprogramming the function of the endothelial cells. Increased amounts of tetraspanin 8 (Tspan8), a proangiogenic protein, were detected in exosomes isolated from peritoneal fluid of patients with CRC [46]. Several miRNA-enriched exosomes promote angiogenesis via HIF-1 downregulation and overexpression of proangiogenic factors [47]. Recently, He et al. suggested that ex-miR-21-5p is involved in angiogenesis and vascular permeability in CRC, by targeting trapped protein 1 (KRIT1) [48]. Notably, Simon et al. have reported a potential EV-mediated mechanism for bevacizumab resistance in glioblastoma. The authors suggested that shedding of bevacizumab at the surface of glioblastoma-derived EVs may be way of cancer cells to resist treatment [49].

Finally, it has been shown that tumor-derived exosomes are loaded with immunosuppressive molecules that can decrease CD4, CD8 and NK cells [50,51,52]. Moreover, exosomal PD-L1 has been reported to confer immunity resistance to cancer cells by inhibiting T-cell activation [53]. Immune checkpoint blockade (nivolumab ± ipilimumab or pembrolizumab) is currently a treatment option for microsatellite instability-high (MSI-H) or mismatch repair deficient (dMMR) mCRC. Infiltrating immune cells represent a pivotal content of the TME and are directly associated with immunotherapeutic effect [54]. Chen et al. found that exosomal circUSP7 promotes resistance to anti-PD1 therapy, by inducing CD8 T-cell dysfunction in non-small cell lung cancer (NSCLC) [55]. Similarly, a recent study showed that miR-424-loaded EVs amplified immune checkpoint blockade resistance, by suppressing the CD28-CD80/86 pathway [56]. On the other hand, selective loading of exosomes with tumor-suppressive miRNAs, such as miR-124 seems to regulate immune responses from CD4 and CD8 T cells, leading to enhanced immunotherapeutic efficacy [57]. Further research concerning the role of exosomal ncRNAs in CRC immunotherapy resistance is anticipated.

## 4. Exosomal ncRNAs in CRC Drug Resistance: Current Evidence

### 4.1. Exosomal miRNAs and CRC Drug Resistance

Exosomal miRNAs (ex-miRNAs) were the first ncRNA subtype to be studied in the field of cancer drug resistance. Huang et al., in 2013, was one of the first to report that, among other RNA classes, miRNAs were the most abundant in human plasma derived exosomes [27]. MicroRNAs (miRNAs) represent a class of small non-coding RNA molecules (18–22 nucleotides long) that bind to the complementary 3′ untranslated region (UTR) of the mRNA and promote mRNA cleavage and degradation. MiRNAs can act as oncogenes or tumor suppressors and they are involved in several biological processes, such as cell proliferation, migration, differentiation, metabolism, invasion, and apoptosis. To date, how exosomes regulate miRNA expression in cancer cells is still under investigation [6,58]. Exosome-mediated transfer of miRNAs promotes genetic exchange among cells and plays an important role in modulating gene expression and cell function of the recipient cells. On this basis, increasing evidence demonstrates that tumor-secreted exosomal miRNAs can also regulate drug resistance in sensitive cells [59,60].

Despite that several studies have investigated the role of miRNAs in CRC drug resistance, only few have focused specifically on the exosomal miRNA profiling and role until now (Table 1). Fluoropyrimidines (especially 5-FU) are an essential element of systemic chemotherapy for CRC in both adjuvant and metastatic settings, and thus 5-FU resistance has been a topic of great interest over the years. In 2014, Akao et al. reported that the secretion of tumor suppressor miRNAs (miR-34a and miR-145) in MVs results in 5-FU resistance, due to their decreased intracellular levels [61]. An association between miR-21 and 5-FU resistance has been also described, via PDCD4 (Programmed cell death 4) downregulation [62]. Interestingly, 5-FU resistance can be reversed by the combined delivery of a miR-21 inhibitor and 5-FU, encapsulated in engineered exosomes [63]. Similarly, Yao et al. found that miR-204-5p increases 5-FU sensitivity via the suppression of target genes, such as RAB22A and Bcl2 [64]. Loss of exosomal transferred miR-200c in 5-FU resistant colon cells was associated with lymphendothelial invasiveness, by regulating the EMT transcription factors ZEB1 and SLUG [65]. Holzner et al. also linked 5-FU resistance with exosomal miR-200 family, via the modulation of resistance of adjacent blood endothelial barriers in vitro [66]. Moreover, miR-196b-5p, was found to enhance stemness and 5-FU resistance via targeting SOCS1 and SOCS3 and activating STAT3 signaling pathway [67].

Oxaliplatin is a widely used compound in CRC management. Liu et al. found that low expression of miR-128-3p in CRC tumors before treatment was significantly associated with poor response to oxaliplatin. In addition, the results showed that exosome-transmitted miR-128-3p could improve the therapeutic outcome in oxaliplatin-resistant CRC. MiR-128-3p exerts its multifactorial role by (i) suppressing EMT, (ii) increasing intracellular drug accumulation and (iii) decreasing drug efflux [68]. Reportedly, ex-miR-46146 modulate resistance through PDCD10 (Programmed cell death 10). More importantly, the authors suggested that PDCD10 overexpression may also reverse oxaliplatin resistance [69]. MiR-208b is another ex-miRNA that has been implicated with oxaliplatin resistance. According to the proposed mechanism, cancer cell-transferred miR-208b to recipient T-cells enhances Treg expansion by targeting programmed cell death factor 4 (PDCD4) and promotes chemoresistance [70]. Gw4869 is a small inhibitor which regulates exosome release; gw4869-induced inhibition of ex-miR-19b secretion was found to be associated with oxaliplatin sensitivity [71]. Moreover, ex-miR-1915-3p upregulation has been negatively correlated with EMT-promoting oncogenes and was proposed as a potential modifier of oxaliplatin resistance [72]. A panel of 4 up- and 20 down-regulated miRNAs, identified in clinical samples from oxaliplatin-resistant patients, were associated with the RNA polymerase II transcription system and were enriched in the PI3K-AKT, AMPK and FoxO signaling pathways [73]. Drug resistance has been also associated with gene demethylation. Tanaka et al. reported that DNA demethylation treatment is correlated with acquisition of epithelial cell-like characteristics. More specifically, ex-miR-200c and ex-miR-141 were suggested as potential biomarkers for mesenchymal–epithelial transition of oxaliplatin-resistant CRC cells [74] while the exosomal expression level of let-7b was associated with the cells’ invasion activity [75]. Last, but not least, another work by the same authors reported that the downregulation of miR-33a-5p and miR-210–3p is correlated with oxaliplatin resistance; a possible explanation implicates the inhibition of several genes related with DNA damage repair mechanisms [76].

Furthermore, several chemoresistance-driven studies in CRC include traditionally used combination regimens, such as FOLFOX. A panel of six exosomal miRNAs has been linked with 5-FU/OX resistance via (i) PI3K-Akt, (ii) FoxO and (iii) autophagy-animal pathways [77]. EMT and cell stemness regulation by ex-miRNAs is also a well-established mechanism for CRC chemoresistance. Hue et al. found that increased expression of miR-92a-3p in CRC tissues activates the Wnt/β-catenin pathway and inhibits mitochondrial apoptosis by directly inhibiting FBXW7 and MOAP1. The authors concluded that miR-92a-3p upregulation was associated with cell stemness, EMT, metastasis and 5-FU/OX resistance in CRC [3]. In line with this observation, exosomes containing miR-210 were found to promote EMT and metastasis in 5-FU and oxaliplatin-treated cells [78]. Finally, it has also been suggested that plasma exosomal miR-125b may act as a biomarker for the early detection of resistance to mFOLFOX6-based chemotherapy; no potential resistance mechanism was reported [79].

The introduction of targeted treatments and immunotherapy has dramatically changed the therapeutic landscape of mCRC, over the last decade. However, even novel targeted therapies eventually fail due to drug resistance. For example, high expression of EV-derived miR-92a was associated with shorter PFS and OS in bevacizumab plus FOLFOX6-treated mCRC patients [80]. Furthermore, Zhao et al. investigated the immune checkpoint inhibitor-induced resistance in CRC. They proposed that tumor-secreted EVs containing miR-424 suppressed the CD28-CD80/86 co-stimulatory pathway in tumor-infiltrating T cells and dendritic cells, leading to immune checkpoint blockade resistance. More importantly, modified tumor-secreted EVs containing knocked down miR-424 enhanced T-cell-mediated antitumor immune response in colorectal cancer tumor models and increased the immune checkpoint blockade response [56].

Finally, Zhang et al. associated methotrexate resistance with exosomal CAF-derived miR-24-3p; miR-24-3p overexpression was reported to enhance resistance of colon cancer cells to MTX by down-regulating the caudal-related homeobox 2/hephaestin (CDX2/HEPH) axis [81].

### 4.2. Exosomal Long ncRNAs and CRC Drug Resistance

Long non-coding RNAs (lncRNAs) represent another widely studied, diverse type of ncRNAs that display complex gene regulatory functions in the cells. Typically, lncRNAs are more than 200 nucleotides long and can act as crucial mediators of a variety of processes, such as cancer development, metastasis and chemoresistance. Moreover, lncRNAs may often regulate gene expression by interacting with miRNAs as ceRNAs (competing endogenous RNAs). Similar to miRNAs, lncRNAs can also be transferred via exosomes and confer distinct functions to recipient cells.

The exosomal lncRNA, CCAL has been correlated with oxaliplatin resistance. More specifically, CCAL activates β-catenin and suppresses apoptosis by directly interacting with mRNA stabilizing protein HuR (human antigen R) [42]. Additionally, ex H19 has been found to mediate oxaliplatin resistance; this lncRNA exerts its actions by activating the β-catenin pathway (serving as a competing endogenous RNA for miR-141) [41]. Moreover, based on the results from a bioinformatics analysis, oxaliplatin or irinotecan resistance is associated with an imbalance between cell proliferation and apoptosis, cell energetic metabolism under hypoxic conditions and angiogenesis. The proposed molecular mechanisms include specific lncRNAs, such as CRNDE, H19, UCA1 and HOTAIR [82]. Recently, another lncRNA, PGM5-AS1 was found to reverse oxaliplatin resistance of CRC cells by acting as a sponge for miR-423-5p. More importantly, the authors suggested that combined delivery of PGM5-AS1 and oxaliplatin by engineered exosomes can reverse drug resistance [83].

Yang et al. reported that cetuximab-resistant CRC cells secrete the exosomal lncRNA-UCA1 and associated its expression with cetuximab efficacy. This study demonstrated that UCA1 acts as a sponge for miR-204-5p, thus upregulating the expression of several target genes; UCA1 was found overexpressed in cetuximab-resistant cancer cells and their exosomes, in the progressive/stable disease group of patients. The authors also reported that exosomes were able to transmit cetuximab resistance from resistant cells to sensitive ones [84]. lncRNA-HOTTIP is upregulated in mitomycin-resistant CRC cells and can confer mitomycin resistance following encapsulation into exosomes; mechanistically, lncRNA-HOTTIP increases the levels of karyopherin subunit alpha 3 (KPNA3) in sensitive cells, by binding to miR-214 [85].

Despite the emerging role of lncRNAs in cancer, to date very few research has focused on exosomal lncRNAs in cancer drug resistance (Table 2). Therefore, further investigation is needed in order to determine the involvement of exosomal lncRNAs in CRC drug resistance.

### 4.3. Exosomal circRNAs and CRC Drug Resistance

CircRNAs comprise another type of bioactive ncRNAs that can be loaded in exosomes. CircRNAs form unique, circular, covalently closed loop structures that are more stable compared to linear RNAs. Of note, circRNAs also display high concentrations and stability in exosomes and thus they are considered as a promising cancer biomarker [86]. Growing evidence suggests that exosomal circRNAs play critical roles in cell proliferation, EMT, metastasis and chemoresistance [87].

Wang et al. reported that hsa_circ_0005963 is positively correlated with resistance to oxaliplatin in CRC. In fact, hsa_circ_0005963 was found to promote glycolysis and induce chemoresistance through miR-122 and PKM2 upregulation [45]. Exosomal circRNA FBXW7 sponges miR-18b-5p, leading to decreased oxaliplatin efflux, EMT inhibition and increased oxaliplatin sensitivity [88]. Furthermore, according to Zhao et al., exosomal circ_0000338 overexpression has been corelated with 5-FU resistance, via direct binding with miR-217 and miR-485-3p [89]. In another study, a total of 105 significantly upregulated and 34 downregulated exosomal circRNAs were identified. Among all circRNAs, circ_0000338 emerged as the most important; interestingly, hsa_circ_0000338 exhibits dual regulatory roles in chemo-resistant CRC, displaying different properties in CRC cells (tumor-suppressive) and CRC exosomes (oncogenic). The authors concluded that exosomal hsa_circ_0000338 is associated with 5-FU and oxaliplatin resistance in CRC [90]. Finally, exosomal transfer of circ_0006174 was found to increase doxorubicin resistance in CRC, by targeting the miR-1205/CCND2 axis [91].

The existing data regarding the role of exosomal circRNAs in CRC drug resistance is limited and further investigation is needed, in order to identify novel resistance-related biomarkers (Table 3).

### 4.4. Exosomal siRNAs and CRC Drug Resistance

Small interfering RNAs (siRNAs) are a class of double-stranded non-coding RNA that exerts its actions through the RNA interference (RNAi) pathway. More specifically, they interfere via targeting and silencing of genes and prevent translation by degrading mRNA after transcription. Nowadays, siRNAs have gained increased scientific interest and research has initiated regarding their potential role in CRC. The effect of siRNA-mediated silencing of vascular endothelial growth factor (VEGF) expression in human colorectal cancer cells has been reported to lead towards decreased tumor proliferation in CRC [92]; nevertheless, their potential use warrants additional research due to safety and efficiency issues [93].

In the present review, only two studies were found to include exosomal siRNAs in CRC drug resistance (Table 4). The first work was conducted by Zhang et al. The authors revealed a rather unconventional mechanism of acquired drug resistance, according to which p-STAT3-containing exosomes contribute to acquired 5-FU resistance in CRC. More specifically, p-STAT3 transferred by exosomes from 5-FU-resistant cells could induce chemotherapy resistance in recipient cells by reducing caspase cascade activation; on the other hand, p-STAT3 inhibition re-sensitized cells to 5-FU [94]. The second identified original research paper showed that iRGD-modified exosomes promote CPT1A (carnitine palmitoyltransferase 1A) downregulation in tumor tissues leading to inhibition of fatty acid oxidation; thus, since fatty acid oxidation plays a vital role in cancer chemoresistance, CPT1A siRNAs could inhibit tumor growth and reverse resistance to oxaliplatin [95].

## 5. Final Remarks and Future Perspectives

Overcoming cancer drug resistance remains a great challenge for clinicians and researchers. In recent years, the tight connection between the TME and drug resistance acquisition by cancer cells has been well established [96]. On this basis, reversion of pharmacological-resistant phenotypes can be achieved by both directly targeting cancer cells and modulating the TME. Moreover, besides the local stroma and microenvironment, tumors also generate the formation of microenvironments (pre-metastatic niches, PMNs) in distant organs, in order to enable tumor localization and survival before they migrate to metastatic sites. Garcia-Mayea et al. proposed an interesting resistance scenario, according to which resistant CSCs in primary tumor sites undergo EMT and are more likely to achieve a favorable PMN [97]. Those local and distant modifications are mediated by cellular crosstalk and exosomes are known to be crucial effectors of intercellular communication. Indeed, accumulating evidence shows that ncRNA transfer by exosomes may alter recipient-cells phenotypes and their response to cancer treatment. These findings suggest that ex-ncRNAs may potentially serve in the quest for drug resistance/response biomarkers in CRC.

Several studies have investigated the potential role of exosomal ncRNAs in CRC chemoresistance. Of all identified studies included in this review, the vast majority included chemotherapy agents (alone or in combination), whereas only two included targeted therapy agents and one ICIs. Based on our results, further research should focus on exosomal transfer of specific ncRNAs and response to other treatment classes approved for CRC, besides chemotherapy. For example, IL-17A has been reported to increase PD-L1 expression through the p65/NRF1/miR-15b-5p axis, promoting resistance to anti-PD-1 therapy; therefore, blocking IL-17A improved the efficacy of anti-PD-1 therapy in MSI stable CRC [98]. The lncRNA LINK-1 was also found to induce PD-1 drug resistance in breast cancer [99]. Additionally, future research could also investigate any possible associations between drug resistance/response to anti-HER2 treatment and exosomal ncRNAs. HER2 blockade combinations have been included as treatment options for HER2 positive mCRC. Similar studies in other tumor types, such as breast cancer, have shown that exosomal transfer of the lncRNA-small nucleolar RNA host gene 14 (SNHG14) induced trastuzumab resistance, via the BCL-2/Bcl-2-associated X pathway; serum exosomal SNHG14 was overexpressed in trastuzumab-resistant patients [100]. Moreover, exosomal miR-567 is implicated with trastuzumab resistance reversion in breast cancer via inhibition of autophagy-related 5 (ATG5) protein [40]. In line with the above, our literature search identified only one article including bevacizumab plus chemotherapy. Tumor angiogenesis is a momentous process in cancer progression and metastasis; the tumor microenvironment comprises several molecules and pathways, such as pro-angiogenic and anti-angiogenic factors. Evidence shows that ncRNAs are also involved in tumor angiogenesis and therefore contribute to chemoresistance. For instance, Jin et al. announced that, among others, lncRNA H19, MEG3, HOTAIR, MALAT1, TUG1 and PVT1 affect the process of angiogenesis [101]. More dedicated studies should be conducted searching the potential role of exosome-mediated resistance towards anti-angiogenic agents in CRC.

Although radiotherapy has been established, especially for rectal cancer, as a mainstay of treatment alongside surgery, the aim of this review was to discuss specifically the role of ex-ncRNAs in drug resistance. However, it should be mentioned that ex-ncRNAs have been also implicated with CRC resistance to radiotherapy. Only two original research articles were found during our research. Exo-miR-19b transfer from CRC to recipient cells, has been linked with enhanced stemness and radioresistance, via FBX downregulation and Wnt/β-catenin pathway activation. These results suggest that miR-19b inhibition could promote CRC cell resensitisation to radiotherapy [102]. Another radioresistance mechanism, implicating ex-miRNAs has been proposed by Chen et al. CAF-derived exosomes were found to enclose miR-93-5p; the latest inhibits radiation-induced apoptosis, possibly by downregulating FOXA1 and upregulating TGFB3. Consequently, targeting ex-miR-93-5p in CAFs may provide a promising opportunity to overcome CRC radioresistance [103].

Besides their potential as drug resistance/response biomarkers, another future application for ex-ncRNAs is associated with their potential as therapeutics. Exosomes have been proposed as a promising nano-delivery vehicle. Indeed, exosomes exhibit unique properties, such as increased stability in body fluids and tissues, biocompatibility, low immunogenicity, and ability to cross barriers, such as the blood–brain barrier (BBB) [104]. Selective loading of exosomes with ncRNAs that could resensitize cancer cells to drugs, may serve to reverse drug resistance. On the other hand, several ncRNAs have been associated with promoting drug resistance; thus, exosome-mediated transfer of ncRNA inhibitors could also help towards re-establishing drug sensitivity. However, despite being a very promising option, the field of engineered exosomes remains in its infancy and warrants further investigation. Interestingly, lncRNAs are reported to be more effective compared to conventional anti-miRNA approaches in terms of exerting anti-drug resistance functions [105]. Another topic that should be addressed and requires additional safety evaluation is the tumorigenic potential of administering engineered exosomes to patients. There is some concern whether isolated tumor-derived exosomes may possess oncogenic factors even after in vitro manipulations and thus, if re-introduced to patients, they may promote tumorigenesis. Despite that Yin et al. reported that mesenchymal stromal cell-derived exosomes have significantly safer profiles compared to tumor-derived exosomes, more research should be conducted in terms of safety [106]. For more details on the potential future application of exosomal ncRNAs in CRC, we recommend referring to Table 1, Table 2, Table 3 and Table 4.

Moreover, although the clinical use of exosomes appears promising, their study faces huge challenges. To date, only a small fraction of ex-ncRNAs has been studied, and the mechanisms of exosomal ncRNAs underlying CRC initiation, progression, metastasis, and drug resistance need to be elucidated in more detail. In addition, the exact recipient cell uptake processes of exosomes remain unclear. Future work should also investigate whether the primary tumor still produces ex-ncRNAs to regulate the metastatic microenvironment, after distant metastasis is developed, and if distant metastasis also releases ex-ncRNAs to formulate metastatic niches in other distant organs. Consequently, an interesting topic to be addressed is how ex-ncRNAs released by metastatic sites regulate the primary TME.

Another limitation relates to the methods used for isolating exosomes. Despite improvements, exosomes’ isolation and purification are still difficult, time-consuming, and laborious. Furthermore, it is very critical to identify reliable exosomal, drug resistance-associated ncRNAs and validate such candidates in large cohorts of samples. Finally, one should keep in mind that a unique ncRNA profile may not be sufficient to serve as an accurate marker of chemosensitivity; instead, the discovery of a ncRNA panel could display improved performance [63].

To sum up, the mechanisms of drug resistance are complex, involving several different pathways. Likewise, a certain ncRNA may modulate different pathways of resistance depending on a plethora of parameters, such as the type of cancer or the type of drug. Another riddle is whether these mechanisms change in different cancer stages or types of chemoresistance (i.e., intrinsic versus acquired) and whether this can be exploited for reversing resistance and improving efficacy of CRC treatment.

## 6. Conclusions

Although treatment advances have improved survival rates in current years, drug resistance is often responsible for treatment failure in CRC. Cancer drug resistance has been implicated with exosomes due their ability to cross biological barriers and provide functional ncRNAs to recipient cells. Despite several challenges, ex-ncRNA signatures may be used as (a) a candidate biomarker for predicting drug resistance and monitoring drug efficacy and (b) a targeted exosomal load to reverse drug desensitization. Further, larger-scale research is needed in order to identify and validate exosomal ncRNAs as therapy monitoring biomarkers in CRC.

## Figures and Tables

**Table 1:** List of exosomal miRNAs involved in CRC chemoresistance or chemosensitivity.

EV/Exosome Content	Expression	Type of EVs	EV Source	Major Findings	Potential Clinical Application	Drug	Ref.
miR-92a-3p	↑	exosomes	CAFs isolated from tissues and serum samples	(i) CAF-derived exosomes transfer miR-92a-3p enhancing cell stemness, EMT, metastasis and chemoresistance (ii) Overexpression of exosomal miR-92a-3p in serum is highly linked with metastasis and chemoresistance	Prognostic value/Therapy monitoring biomarker	5-FU/OX	[3]
miR-424	↑	EVs	Tumor-derived EVs (human and mouse tumor samples, cell lines, human colorectal cancer organoids, and mouse models)	(i) EVs containing miR-424 were associated with immune checkpoint blockade resistance (ii) Modified tumor-secreted EVs ↓miR-424 boost the immune checkpoint blockade efficacy in late-stage disease	Therapeutic target	ICIs	[56]
miR-34a and miR-145	↑	EVs	Cell lines (DLD-1 and DLD-1/5-FU)	Selected secretion of miR-34a and miR-145, following 5-FU exposure results in decreased intracellular levels of tumor suppressor miRNAs (such as miR-34a and miR-145)	Therapeutic potential/Therapy monitoring biomarker	5-FU	[61]
miR-21	↑	exosomes	Cell lines (HT29, T84, and LS174)	targeted secretion of exosomes carrying miR-21, may represent an effective anti-cancer strategy	Therapeutic target	5-FU	[62]
miR-21 inhibitor	-	purified engineered exosomes	Cell lines (THLG-293T, LG-293T and GFP-293T)	miR-21i and 5-FU co-delivery reverses drug resistance and enhances cytotoxicity	Therapeutic potential	5-FU	[63]
miR-204-5p	↑	exosomes	Cell lines (293T-GFP and 293T-miR-204 cells)	Exosomal miR-204-5p induces apoptosis, inhibits cell proliferation, and increases chemosensitivity	Therapeutic potential	5-FU	[64]
miR-200 family	↓	exosomes	Cell line (CCL227 cells)	Loss of miR-200 family is associated with lymphendothelial invasiveness and chemoresistance	Prognostic value/Therapeutic potential	5-FU	[65]
miR200	↓	exosomes	Cell lines (CCL227)	Exosomes devoid of miR200 in 5-FU resistant cells are associated with the induction of the metastatic process	N/A	5-FU	[66]
miR-196b-5p	↑	exosomes	Serum	(i) miR-196b-5p overexpression is associated with poor survival (ii) Silencing miR-196b-5p enhances 5-FU-induced apoptosis (iii) miR-196b-5p downregulation reverses chemoresistance	Prognostic value/therapy monitoring biomarker/therapeutic target	5-FU	[67]
miR-128-3p	↑	exosomes	Cell line [normal intestinal epithelial (FHC) cells]	(i) miR-128-3p suppresses EMT and increases intracellular drug accumulation (ii) miR-128-3p downregulation is associated with poor response in advanced CRC (iii) miR-128-3p overexpression inhibits EMT and decreased drug efflux	Prognostic value/therapy monitoring biomarker/therapeutic potential	OX	[68]
miR-46146	↑	exosomes	Cell lines	Exosomal miR-46146 promotes resistance	Therapy monitoring biomarker/therapeutic target	OX	[69]
miR-208b	↑	exosomes	Cell lines (NCM460, SW480, SW480-OXA cells)	(i) Exosomal miR-208b is overexpressed in oxaliplatin resistant cell lines (ii) Exosomal miR-208b is delivered into recipient T cells, promoting Tregs	Predictive biomarker/therapeutic target	OX	[70]
miR-19b	↑	exosomes	Cell line (SW480 cells)	Suppressing exosomal miR-19b release leads to increased sensitivity	Therapeutic potential	OX	[71]
miR-1915-3p	↑	EVs	Non-tumorigenic intestinal cell line (FHC)	Exosomal delivery of miR-1915-3p improves chemotherapeutic effect	Therapeutic potential	OX	[72]
miR-100, miR-92a, miR-16, miR-30e, miR-144-5p, and let-7i	↑	exosomes	Plasma	A panel of 6 exosomal miRNAs can significantly distinguish chemoresistant from chemosensitive patients	Therapy monitoring biomarker/therapeutic potential	OX	[73]
Let 7b	↑	exosomes	Cell lines (SW480, SW620, and SW620/OxR).	(i) Oxaliplatin resistance may inhibit the incorporation of let-7a, let-7b, and let-7g into exosomes (ii) Exosomal let-7a, let-7b, and let-7g are downregulated in resistant cells	Therapy monitoring biomarker	OX	[74]
miR-200c and miR-141	↑	exosomes	Cell lines (SW620/OxR, SW480, SW620)	Up-regulated miR-200c and miR-141 following decitabine treatment was associated with mesenchymal-epithelial transition of oxaliplatin -resistant CRC cells	Predictive biomarker	OX	[75]
miR-33a-5p, miR-210–3p	↓	small EVs (sEVs)	Cell lines (SW620, SW480 and SW620-OxR cells)	miR-33a-5p and miR-210–3p are under-expressed in oxaliplatin-resistant cells	Therapy monitoring biomarker	OX	[76]
miR-21-5p, miR-1246, miR-1229-5p, miR-135b, miR-425 and miR-96-5p	↑	exosomes	Serum	This panel of exosomal miRNAs could significantly distinguish chemotherapy-resistant patients	Therapy monitoring biomarker/therapeutic target	5-FU/OX	[77]
miR-210	↑	exosomes	Cell line (HCT-8 cells)	(i) Exosomal miR-210 is associated with significantly lower chemosensitivity (ii) Exosomal miR-210 promotes EMT and metastasis	Predictive biomarker/therapeutic target	5-FU, FOLFOX-like treatment (5FU + Oxaliplatin)	[78]
miR-125b	↑	exosomes	Plasma	(i) Exosomal miR-125b has been correlated with chemoresistance (ii) Exosomal miR-125b before chemotherapy is a predictive biomarker for PFS	Predictive biomarker/therapy monitoring biomarker	mFOLFOX6	[79]
miR-92a	↑	EVs	Serum EVs isolated from 44 mCRC patients	(i) Overexpression of miR-92a was associated with shorter PFS (close but not statistically significant)	Further investigation is needed	Bevacizumab + FOLFOX-6m (5FU,LV,OX)	[80]
miRNA-24-3p	↑	exosomes	miR-24-3p inhibitor-treated CAFs (CRC tissues and cells)	(i) CAF-derived exosomal miR-24-3p was associated with chemoresistance (ii) CAF-derived exosomal miR-24-3p inhibitor enhanced cell apoptosis in vitro and inhibited tumor growth in vivo	Therapy monitoring biomarker/therapeutic target	MTX	[81]

EVs: extracellular vesicles; CRC: colorectal cancer; mCRC: metastatic colorectal cancer; CAF: cancer-associated fibroblast; 5-FU: 5-Fluorouracil; OX: oxaliplatin; LV: leucovorin; ICIs: immune checkpoint inhibitors; MTX: methotrexate; EMT: epithelial–mesenchymal transition; PFS: progression-free survival; Tregs: regulatory T cells; ↑: increased; ↓: decreased.

**Table 2:** List of exosomal lncRNAs involved in CRC chemoresistance or chemosensitivity.

EV/Exosome Content	Expression	Type of EVs	EV Source	Major Finding	Potential (Clinical) Application	Drug	Ref.
H19	↑	exosomes	Tumor tissues, normal tissues and cell lines (HCT116 and SW480)	(i) H19 is overexpressed in CAF-derived exosomes (ii) CAFs-derived exosomal H19 promotes stemness and chemoresistance	Prognostic value/therapeutic target	OX	[41]
CCAL	↑	exosomes	CAFs isolated from human CRC tissues and NFs	CAF-derived exosomal CCAL is transferred to cancer cells inhibiting apoptosis and conferring chemoresistance	Therapeutic monitoring/therapeutic target	OX	[42]
CRNDE, H19, UCA1 and HOTAIR	↑	bioinformatic analysis	-	integrated analysis showed that several differentially expressed genes of lncRNAs are components of the extracellular exosomes	Further investigation is needed	OΧ, IRI	[82]
PGM5-AS1	↓	exosomes	Tumor tissues	(i) PGM5-AS1 hinders proliferation, metastasis, and acquired oxaliplatin resistance of colon cancer cells (ii) Exosomes encapsulating oxaliplatin and PGM5-AS1 can reverse drug resistance	Therapeutic target	OX	[83]
UCA1	↑	exosomes	Serum samples and cell lines (Caco2)	(i) UCA1 was overexpressed in cetuximab-resistant cancer cells and their exosomes (ii) Circulating exosomal UCA1 was significantly overexpressed in progressive disease/stable disease than in the partial response/complete response patients. (iii) Exosomal transfer of UCA1 transmits cetuximab resistance from resistant cells to sensitive ones	Therapy monitoring biomarker/prognostic biomarker/therapeutic target	cetuximab	[84]
HOTTIP	↑	EVs	Serum and cell lines (HCT116, SW620, FHC, LoVo, HT29, SW480, SW1116, and Caco2)	(i) HOTTIP was overexpressed in mitomycin-resistant CRC cells and its inhibition reduced resistance (ii) EV-transferred HOTTIP from mitomycin-resistant cells contributes to mitomycin resistance	Predictive value/therapeutic target	mitomycin	[85]

EVs: extracellular vesicles; CRC: colorectal cancer; CAF: cancer-associated fibroblast; OX: oxaliplatin; IRI: irinotecan; NF: normal fibroblasts; ↑: increased; ↓: decreased.

**Table 3:** List of exosomal circRNAs involved in CRC chemoresistance or chemosensitivity.

EV/Exosome Content	Expression	Type of EVs	EV Source	Major Finding	Potential (Clinical) Application	Drug	Ref.
hsa_circ_0005963	↑	exosomes	Serum and cell lines (SW480, HCT116, and HEK293)	(i) Circular RNA hsa_circ_0005963 was associated with chemoresistance (ii) Exosomal hsa_circ_0005963 from oxaliplatin-resistant cells confers resistance to sensitive ones (ii) Exosomal transport of si-ciRS-122 suppresses glycolysis and reverses resistance	Therapy monitoring biomarker/therapeutic target	OX	[45]
FBXW7	↓	exosomes	FHC cell culture	(i) circ-FBXW7 was decreased in oxaliplatin-resistant CRC patients and cells. (ii) Exosomal circ-FBXW7 delivery can improve chemoresistance	Therapeutic potential	OX	[88]
circ_0000338	↑	exosomes	Cell lines (SW480/5-FU and HCT116/5-FU)	(i) Circ_0000338 was upregulated in 5-FU-resistant CRC tissues and cells, and its knockdown reversed 5-FU resistance (ii) Exosome-mediated delivery of circ_0000338 confers chemoresistance to sensitive cells	Therapy monitoring biomarker/therapeutic target	5-FU	[89]
hsa_circ_0032883, hsa_circ_0066629, hsa_circ_0002039, and hsa_circ_0000338	↑	exosomes	FOLFOX-resistant HCT116-R cells	(i) 105 upregulated and 34 downregulated circRNAs in exosomes from FOLFOX-resistant cells (ii) Drug resistance can be conferred from resistant cells to sensitive ones via exosomes (ii) Exosomal hsa_circ_0000338 was differentially upregulated in chemoresistant cells	Therapy monitoring biomarker	5-FU, OX	[90]
circ_0006174	↑	exosomes	Tissue samples	(i) Circ_0006174 was overexpressed in doxorubicin-resistant cells and its downregulation reversed resistance and metastatic potential (ii) Exosomal transfer of circ_0006174 enhances chemoresistance	Therapy monitoring biomarker/therapeutic target	DOX	[91]

EVs: extracellular vesicles; CRC: colorectal cancer; 5-FU: 5-Fluorouracil; OX: oxaliplatin; DOX: doxorubicin; FOLFOX: 5-Fluorouracil, leucovorin, oxaliplatin; ↑: increased; ↓: decreased.

**Table 4:** List of exosomal siRNAs involved in CRC chemoresistance or chemosensitivity.

EV/Exosome Content	Expression	Type of EVs	EV Source	Major Finding	Potential (Clinical) Application	Drug	Ref.
GSTP1 and STAT3 siRNAs	-	exosomes	Cell lines (RKO/R and RKO/P)	p-STAT3 transferred by exosomes from 5-FU-resistant cells confers chemoresistance	Therapeutic potential	5-FU	[94]
CPT1A siRNA	-	exosomes	Tumor Tissue	(i) Silencing CPT1A by siRNA could reverse chemoresistance and combined delivery of oxaliplatin with CPT1A inhibitor promotes apoptosis and proliferation (ii) iRGD-engineered exosomes with siCPT1A suppress fatty acid oxidation and thus reverse chemoresistance	Therapeutic potential	OX	[95]

EVs: extracellular vesicles; 5-FU: 5-Fluorouracil; OX: oxaliplatin; GSTP1: glutathione S-transferase Pi 1; STAT3: signal transducer and activator of transcription 3; pSTAT3: phospho-STAT3; CPT1A: carnitine palmitoyltransferase 1A.

## Data Availability

Not applicable.
